# From Inflammation to Neuroplasticity: Molecular Mechanisms of Pain in Acute, Recurrent and Chronic Pancreatitis

**DOI:** 10.3390/ijms27146383

**Published:** 2026-07-17

**Authors:** Cristina Patoni, Stella Ioana Popescu, Christopher Pavel, Marius Nicolae Popescu, Cristian Gheorghe

**Affiliations:** 1 Doctoral School, Carol Davila University of Medicine and Pharmacy, 020021 Bucharest, Romania; cristina.patoni@drd.umfcd.ro (C.P.); christopher.pavel@gmail.com (C.P.); marius.popescu@umfcd.ro (M.N.P.); drcgheorghe@gmail.com (C.G.); 2 Central Military Emergency University Hospital, 010825 Bucharest, Romania; 3 Department of Gastroenterology, Clinical Emergency Hospital of Bucharest, 014461 Bucharest, Romania; 4 Department of Physical and Rehabilitation Medicine, Elias Emergency University Hospital, 011461 Bucharest, Romania; 5 Gastroenterology Department, Fundeni Clinical Institute, 022328 Bucharest, Romania

**Keywords:** pancreatic pain, acute pancreatitis, recurrent pancreatitis, chronic pancreatitis, neuroinflammation

## Abstract

Pain is the most significant and debilitating symptom throughout all stages of pancreatitis, yet the precise mechanisms responsible for it remain only partially understood, and current pain relief methods often prove insufficient. To gain a clearer understanding, it is crucial to consider pancreatitis not as a singular condition but as comprising three distinct clinical types: acute pancreatitis (AP), recurrent acute pancreatitis (RAP), and chronic pancreatitis (CP), each of which may involve different pathways for pain perception. Notably, RAP has historically been overlooked as a distinct entity, resulting in a paucity of phenotype-specific data regarding its pain mechanisms. At the molecular level, pain in pancreatitis involves peripheral and central sensitization, neuroinflammatory signaling, and structural neural remodeling, processes driven by mediators such as substance P, CGRP, NGF, TRP channels, and pro-inflammatory cytokines. However, these mechanisms have been studied largely in isolation and within single phenotypes, leaving a critical gap in our understanding of how they evolve and interact across the full disease spectrum. This review aims to establish a comprehensive framework detailing the molecular mechanisms responsible for pain in all three pancreatitis phenotypes, with a particular focus on RAP as a less-studied clinical condition and to identify phenotype-specific targets for therapeutic development.

## 1. Introduction

Pancreatitis represents a spectrum of inflammatory disorders of the pancreas, ranging from acute self-limited episodes to recurrent attacks and progressive chronic disease. Across this continuum, pain remains the dominant clinical feature, profoundly impacting quality of life, driving healthcare utilization, and often proving refractory to conventional analgesic strategies [1].

Although much has been done to improve our understanding of pain pathophysiology in pancreatitis, up to 35% of patients undergoing surgery for chronic pancreatitis (CP) may experience persistent or recurrent pain [2], highlighting that there is an underlying mechanism of pancreatic pain sustained by neuroplastic changes in the nervous system.

Unlike most visceral pain syndromes, pain in pancreatitis cannot be reduced to a single pathophysiological mechanism or a single disease stage. It emerges from the convergence of inflammatory, neuroimmune, neuropathic, and central sensitization pathways that interact, amplify one another, and evolve over time [3]. At the peripheral level, cytokines, proteases, and nociceptor-activating mediators initiate the pain signal. At the neural level, intrapancreatic nerves undergo structural and functional remodeling that sustains and intensifies the signal long after the initial inflammatory trigger has resolved. At the central level, the spinal cord and brain are reorganized, becoming not merely recipients of pain but active generators of pain [4,5].

Yet, despite growing evidence for each of these individual mechanisms, the existing literature has largely studied them in isolation and each mechanism predominantly explored in the context of CP alone, with acute pancreatitis (AP) and recurrent acute pancreatitis (RAP) stages receiving far less attention. The result is a fragmented picture: a collection of well-described pieces that have not yet been assembled into a coherent mechanistic narrative. Understanding these interconnected mechanisms is essential for developing personalized, mechanism-based treatment strategies for pancreatic pain.

Furthermore, most recently, research has expanded to encompass epigenetic regulation [6] and the psychological dimensions of chronic pain, creating opportunities for future investigation [7].

A literature search was performed in PubMed and Scopus through May 2026, with no restriction on publication start date, using terms related to “pain mechanisms”, “neuroinflammation”, and “central sensitization” combined with “pancreatitis”, “acute pancreatitis”, “recurrent acute pancreatitis” and “chronic pancreatitis”. Given the narrative, mechanism-focused scope of this review, study selection followed expert-based screening rather than a formal systematic protocol. Both preclinical (animal and in vitro) and clinical (human) studies were eligible for inclusion. Titles and abstracts were screened for direct relevance to molecular, cellular, or clinical evidence on nociceptive signaling, sensitization, or neural remodeling in pancreatitis; full texts were retrieved for studies meeting this criterion. Articles addressing pancreatitis without a specific mechanistic or clinical link to pain, or with only marginal relevance to the review’s scope, were excluded. This approach prioritized depth and mechanistic coherence over exhaustive coverage, consistent with the aims of a narrative synthesis. We aimed to synthesize the evolution of the field, connect findings across studies, and propose a coherent classification framework for pain mechanisms in pancreatitis. By organizing mechanisms into categories such as inflammatory, nociceptor, neuroimmune, neural remodeling, and central, we provide a framework for understanding how these processes amplify one another and how interventions might be rationally targeted.

**Figure 1:**
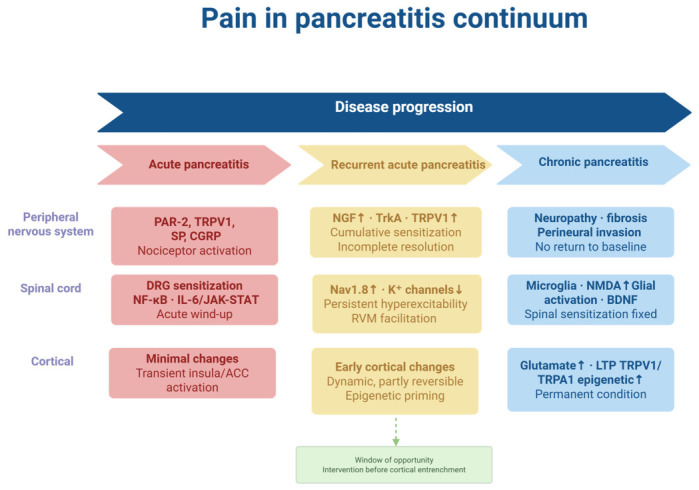
Pain in pancreatitis continuum. Abbreviations: PAR-2, protease-activated receptor 2; TRPV1, transient receptor potential vanilloid 1; SP, substance P; CGRP, calcitonin gene-related peptide; NGF, nerve growth factor; TrkA, tropomyosin receptor kinase A; DRG, dorsal root ganglion; NF-κB, nuclear factor kappa B; Nav1.8, voltage-gated sodium channel 1.8; RVM, rostral ventromedial medulla; NMDA, N-methyl-D-aspartate; BDNF, brain-derived neurotrophic factor; LTP, long-term potentiation; ACC, anterior cingulate cortex. Created in BioRender. Patoni, C. (2026) https://BioRender.com/d5ea069 (accessed on 14 July 2026).

We specifically focus on the less-explored phenotype of recurrent pancreatitis and emphasize recent findings that pave the way for new therapeutic strategies. We propose a stage-dependent, molecular-to-central model of pain evolution across pancreatic continuum (Figure 1).

## 2. Pain Mechanisms in Acute Pancreatitis: The Peripheral Inflammatory Origin

### 2.1. Acinar Injury and PAR-2 Activation as the Initiating Nociceptive Signal

The earliest molecular event that links pancreatic injury to pain is the premature intracellular activation of trypsinogen within acinar cells. Under physiological conditions, trypsinogen activation in the pancreatic duct is tightly regulated by serine protease inhibitor Kazal type 1 (SPINK1). However, this regulation can fail due to mechanical obstruction, hyperstimulation, or direct cellular stress. After the control fails, active trypsin accumulates within and around acinar cells, gaining access to the interstitium and, critically, to the sensory nerve terminals that innervate pancreatic tissue via the splanchnic nerves [8,9].

Trypsin activates protease-activated receptor 2 (PAR-2), a G-protein-coupled receptor expressed at high density on pancreatic acinar and ductal cells and, importantly, on pancreatic sensory nerve fibers. The nociceptive consequence of this interaction is direct and measurable. Intraductal infusion of trypsin at subinflammatory concentrations produces a behavioral nociceptive response. Additionally, it leads to dose-dependent activation of spinal neurons, as measured by Fos immunoreactivity in thoracic dorsal horn segments. This establishes that trypsin-PAR-2 signaling on sensory afferents represents a nociceptive signal that is independent of evident tissue inflammation [10]. This finding was extended by demonstrating the presence of inhibitor-resistant trypsin isoforms. Specifically, human trypsin IV and rat P23 are resistant to pancreatic trypsin inhibitors. These isoforms remain active even when conventional isoforms are neutralized. They produce more robust nociceptive activation and some inflammatory endpoints. Both of these effects are blocked by the broad-spectrum trypsin inhibitor melagatran [11].

Protein PAR-2, which is found on sensory neurons, does not function independently. Once it is cleaved by trypsin, PAR-2 starts a signaling cascade involving two enzymes, protein kinase Cε (PKCε) and protein kinase A (PKA). This process leads to the phosphorylation and increased sensitivity of the co-expressed transient receptor potential vanilloid 1 (TRPV1) channel, lowering its activation threshold and thereby amplifying the nociceptive response to subsequent stimuli [12]. The interaction between PAR-2 and TRPV1 is a crucial molecular transition from protease activation to prolonged ion channel sensitization. Additionally, the same PAR-2 signaling pathway enhances the sensitivity of TRPA1 channels, broadening the spectrum of nociceptive stimuli that the sensory neuron can detect [13].

It should be noted that PAR-2’s role in pancreatitis is not unidimensional. In secretagogue-induced models, PAR-2 activation appears to exert a degree of local protection. This protection occurs through the inhibition of ERK1/2 nuclear translocation in acinar cells. In contrast, in bile salt-induced pancreatitis, PAR-2 contributes to worsening the condition. This worsening is linked to the dysregulation of acinar calcium signaling [14].

This model-dependence highlights the complexity of PAR-2 biology and emphasizes that its role in nociception, acting on sensory nerve terminals, is different from its effects on parenchymal tissue and should be understood as such. These findings are derived predominantly from rodent models; the species-specific limitations of this evidence base, including the current absence of direct human data on trypsin-PAR-2-TRPV1 signaling in pancreatitis, are discussed in Section 5.

### 2.2. TRPV1, Bradykinin Receptors, and Early Neuropeptide Release: Primary Transduction

TRPV1 is known to exist alongside two key neuropeptides: substance P (SP) and calcitonin gene-related peptide (CGRP) [15]. Studies using a rat model of L-arginine-induced pancreatitis have demonstrated that activating TRPV1 significantly enhances pain-related responses. Specifically, activation of TRPV1 resulted in a twelve-fold increase in c-fos expression, a marker for neural activity, in the spinal dorsal horn neurons at the T10 level. Furthermore, it also triggered a threefold rise in spontaneous abdominal contractions, which serve as indicators of referred pain. Importantly, both of these outcomes were entirely eliminated with the systemic administration of the TRPV1 antagonist capsazepine, highlighting the essential role of TRPV1 in mediating these pain responses. Intrathecal, but not systemic, administration of CGRP and SP receptor antagonists also attenuated spinal Fos expression, confirming that the nociceptive signal is carried centrally by neuropeptide release downstream of TRPV1 activation [15]. The TRPV1 channel plays a key role in the release of SP during caerulein-induced pancreatitis. This was demonstrated by observing the internalization of neurokinin-1 receptors (NK1R) in spinal dorsal horn neurons, which serves as a reliable in vivo measure of SP release. Notably, the response was significantly diminished when the TRPV1 antagonist capsazepine was used [16].

Pancreatic inflammation significantly upregulates both TRPV1 and the co-expressed TRPA1 channel in nodose and dorsal root ganglion (DRG) neurons innervating the pancreas. These two channels work synergistically, as evidenced by the fact that combined antagonism of TRPV1/TRPA1 results in a greater reduction in pain behaviors and pancreatic inflammation indices than when either antagonist is used alone [17]. Additionally, neutrophil elastase, released during the inflammatory response, activates PAR-2 and the related TRPV4 channel through a distinct protease-dependent pathway, further expanding the ensemble of nociceptive signals converging on pancreatic sensory afferents [18].

Moreover, the pH of the inflammatory microenvironment provides an additional activation signal for TRPV1. Low extracellular pH, generated by ischemia, cell necrosis, and metabolic acidosis during acute pancreatitis, activates TRPV1 at physiological temperatures through proton-mediated reduction of its thermal activation threshold. This pH-sensitive neurogenic pathway has been directly demonstrated in a post-ERCP pancreatitis model, where the acidity of contrast media was sufficient to activate TRPV1 on C and Aδ fibers and modulate disease severity, an effect reversed by increasing contrast pH or ablating sensory neurons with resiniferatoxin [19].

Concomitant with PAR-2 activation and TRPV1 channel signaling, there is one more pathway involved in the same group of primary afferent neurons, related to bradykinin receptors. Bradykinin, produced locally via the kallikrein-kinin system activated by pancreatic proteases and vascular injury [20], interacts with B1 and B2 receptors found on the same nociceptor population [21]. Under normal conditions, B2 receptors are predominant, but during prolonged inflammation, B1 receptors are also engaged [22]. Bradykinin sensitizes TRPV1 through Gq/11-PLC-PKC-dependent signaling, reducing the thermal activation threshold of TRPV1 to below physiological temperatures [23], thereby enhancing the nociceptive response to stimuli already present in the inflamed tissue. This results in a lowered-threshold state where nociceptors, which would otherwise remain inactive, are recruited into ongoing pain signaling.

### 2.3. Neurogenic Inflammation: Substance P, CGRP, and Neuroimmune Crosstalk

After activation, pancreatic sensory neurons do not just send pain signals to the central nervous system. They also release neuropeptides from their peripheral ends, turning the afferent neuron into an active participant in local tissue inflammation. This two-way signaling, known as neurogenic inflammation, establishes a self-amplifying cycle between the nervous system and the immune response that prolongs and exacerbates the acute pain state. Experimental findings show that activation of primary sensory nerves occurs simultaneously with the initial inflammatory response in acute pancreatitis, and that the secretion of proinflammatory neuropeptides enhances and perpetuates disease progression rather than merely being a passive result of tissue damage. [8,16].

SP and CGRP are both found in the same sensory C fibers and are released together when TRPV1 is activated [15]. Their roles complement each other: SP, acting through NK1R, causes plasma extravasation and granulocyte infiltration from postcapillary venules, while CGRP promotes arteriolar dilation via the calcitonin-like receptor and receptor activity-modifying protein 1 (RAMP1) [24]. Together, they contribute to the edema, immune cell infiltration, and vascular changes seen in pancreatitis. The importance of SP in this context was demonstrated in a mouse study with caerulein-induced disease, in which genetic deletion of NK1R produced marked reductions in hyperamylasemia, pancreatic neutrophil infiltration, acinar cell necrosis, and pancreatitis-associated lung injury. This demonstrates that substance P-NK1R signaling is essential for the full severity of the disease [8].

Research indicates that NK1R is significantly involved in the perception of pain. Scientists discovered that administering an NK1R antagonist to rats suffering from pancreatitis lessened pain-related behaviors, suggesting that the SP-NK1R signaling pathway contributes to the persistence of pancreatic pain, extending beyond inflammation [25]. In the dorsal horn of the spinal cord, the release of SP leads to the internalization of NK1R receptors in specific neurons, indicating the presence of persistent pain signals [26]. Additionally, CGRP enhances this effect by inhibiting SP degradation by neutral endopeptidase [27] and by directly stimulating SP release from primary spinal afferent neurons [15,28].

Neurogenic inflammation in acute pancreatitis is thus bidirectional: immune cell activation releases mediators that sensitize nociceptors, and nociceptors release neuropeptides that amplify immune cell recruitment. The neuroimmune crosstalk extends further through the action of nerve growth factor (NGF), whose expression is upregulated in pancreatic tissue during acute pancreatitis [29]. NGF interacts with its high-affinity receptor, TrkA, located on sensory nerve endings. Once phosphorylated, TrkA initiates retrograde signaling to DRG cell bodies, leading to an increase in the expression of CGRP and preprotachykinin (PPT-A) mRNA. In a rat model of necrotizing pancreatitis, the occurrence of referred mechanical hypersensitivity was directly linked to the phosphorylation of pancreatic TrkA and the upregulation of neuropeptides in the DRG. This hypersensitivity was suppressed by the tyrosine kinase inhibitor k252a, highlighting the role of the NGF-TrkA pathway in mediating peripheral sensitization that results in referred hyperalgesia [30].

### 2.4. NF-κB/MAPK-Driven Cytokine Amplification and Peripheral Sensitization

In the moments following acinar injury, a concurrent and overlapping transcriptional process occurs in acinar cells, macrophages, and infiltrating immune cells. Among the initial events in experimental pancreatitis is the activation of Nuclear factor-kappa B (NF-κB), which becomes evident within 15–30 min of supramaximal cerulein stimulation in acinar cells, even before any histological evidence of cellular damage is visible. This activation triggers the production of a series of pro-inflammatory cytokines, such as TNF-α, IL-1β, IL-6, and the chemokine IL-8 [31,32]. In a prospective study involving human subjects, researchers investigated cytokine trajectories in patients suffering from acute pancreatitis. The study revealed that in severe cases, the proinflammatory response is an early event. From the moment of diagnosis, levels of IL-6, IL-8, and angiopoietin-2 were significantly elevated compared to those in mild and moderate cases. This finding underscores that the cytokine storm initiates at the beginning of the disease, rather than being a subsequent effect of necrosis [33]. Serial measurements in patients showed that IL-1β, IL-6, IL-8, and TNF-α evolved differently across severity grades in the first 48 h, with IL-1β and IL-6 most strongly associated with severe disease [34].

Cytokines function not only as systemic mediators in the inflammatory response but also directly sensitize nociceptors. IL-1β and TNF-α reduce the activation threshold of TRPV1 and voltage-gated sodium channels via both prostaglandin-dependent and independent pathways. Additionally, they activate p38 MAPK in DRG neurons, a kinase that, when activated, elevates TRPV1 protein levels in the peripheral terminals of nociceptors through post-transcriptional mechanisms, thus contributing to heat hypersensitivity [35]. IL-6 levels have a peak within the first 24 h after the onset of severe illness and then decrease thereafter [33]. This cytokine operates through the JAK/STAT3 signaling pathway to make primary sensory pain receptors more sensitive. Vardanyan et al. found that IL-6 was significantly elevated in the thoracic dorsal root ganglia of rats experiencing experimental pancreatitis. Blocking the IL-6 receptor signaling was able to reverse the abdominal hypersensitivity caused by pancreatitis, affecting the peripheral nervous system rather than the central nervous system. This indicates that IL-6 in the dorsal root ganglia plays a critical role in sensitizing pain receptors [36].

This is consistent with the broader pain literature demonstrating that, across multiple inflammatory and neuropathic pain models, IL-6 promotes mechanical allodynia and thermal hyperalgesia through JAK/STAT3-dependent upregulation of pro-nociceptive ion channels in sensory neurons, effects reversed by IL-6 neutralizing antibodies [37]. In the context of AP, NF-κB is also activated within DRG neurons themselves, where it contributes to nociceptive sensitization at the sensory neuron cell body, not just at the peripheral terminal. In a rat model, nitric oxide produced during AP specifically activated the NF-κB pathway within DRGs and simultaneously reduced kappa opioid receptor expression, resulting in a pro-nociceptive state characterized by heightened excitatory signaling and diminished endogenous analgesic tone [38].

The simultaneous action of cytokines at both the inflamed tissue and DRG has significant implications for treatment strategies. These findings suggest that interventions targeting only peripheral inflammation may not effectively interrupt the centrally directed sensitization driven by cytokine effects on sensory neuron gene expression.

### 2.5. Oxidative Stress as an Independent Amplifier of Nociceptive Signaling

Reactive oxygen species (ROS) generated during AP form a mechanistically distinct, yet functionally convergent amplification pathway for nociceptive signaling. During acute pancreatic inflammation, ROS are produced from multiple sources. The primary enzymatic source is NADPH oxidase (NOX)-dependent superoxide generation. In experimental pancreatitis, NOX expression is upregulated, and pharmacological inhibition of NOX has been shown to reduce pancreatic injury. In addition to NOX, oxidant flux is also increased due to activation of xanthine oxidase and dysfunction of the mitochondrial respiratory chain [39,40]. Collectively, this oxidative burst exceeds local antioxidant defenses, resulting in depletion of glutathione and accumulation of lipid peroxidation products in pancreatic tissue [40,41].

The nociceptive effects of this oxidative stress occur through at least two different but complementary mechanisms. Firstly, ROS directly enhance the sensitivity of TRPV1. They do this by covalently modifying intracellular cysteine residues. This oxidative sensitization can be observed in excised inside-out membrane patches and is reversible with reducing agents. Additionally, it works synergistically with kinase-mediated and proton-mediated sensitization. It can also restore agonist sensitivity to receptors that have become desensitized from prolonged exposure to capsaicin. These findings suggest that oxidative modification creates a qualitatively unique and sustained sensitized state [42]. Second, lipid peroxidation products generated during oxidative stress, particularly 4-hydroxynonenal (4-HNE), activate TRPA1 channels on the same nociceptor population through covalent modification of cysteine residues (Cys621, Cys641, Cys665), generating additional pain and neurogenic inflammation independently of TRPV1 activation [43,44].

A third mechanism links oxidative stress to the sensitization of nociceptors at the transcriptional level. In sensory ganglia, excessive ROS generated in response to peripheral inflammation can directly activate TRPA1 and increase its expression. In a primary research study by Zhang et al., it was shown that during periods of peak inflammatory hyperalgesia, prolonged accumulation of ROS occurs within the sensory ganglia. When intraganglionic ROS were scavenged, mechanical hyperalgesia was diminished, and blocking TRPA1 in the ganglia produced similar results. Notably, the introduction of ROS into the ganglion led to the development of pain behaviors and an upregulation of TRPA1 expression, highlighting intraganglionic ROS as a key link between peripheral inflammation and the transcriptional reprogramming of pro-nociceptive channels [45]. In addition, oxidative stress disrupts the function of DNA methyltransferases (DNMTs), leading to widespread ganglionic hypomethylation. This disruption causes a decrease in DNMT3a-mediated methylation at the TRPV1 and TRPA1 promoters, which in turn results in their increased transcriptional activity [45,46]. This epigenetic process is especially significant from the perspective of chronification: even after the acute inflammatory event subsides and ROS levels return to normal, the modified transcriptional profile of the nociceptor may remain, forming a molecular link between the initial episode and the sensitized state that predisposes toward recurrent pain.

Anoctamin 1 (ANO1/TMEM16A), a calcium-activated chloride channel found in small-diameter DRG neurons, serves as an additional amplification point in this pathway. Takayama et al. showed that ANO1 is directly linked to TRPV1 on the cell membrane: the influx of calcium through activated TRPV1 triggers ANO1, leading to a chloride efflux-driven depolarization that boosts action potential firing and reduces the threshold for future activation [47]. The removal of ANO1 in DRG neurons reduces both thermal hyperalgesia and mechanical allodynia in models of inflammatory and nerve injury-induced pain, indicating that ANO1 enhances nociceptor activation instead of just responding to it [48].

Overall, oxidative stress is not simply a consequence of the inflammatory cascade; it actively encodes the inflammatory state within the molecular structure of nociceptors through processes such as channel sensitization, neuropeptide-driven neurogenic loops, and epigenetic reprogramming. These influences extend beyond the initial episode and may set the stage for peripheral sensitization, which, with repeated episodes, can evolve into more severe and treatment-resistant pain types characteristic of chronic diseases. Longitudinal data from quantitative sensory testing in patients with recurrent acute and chronic pancreatitis reveal that pain processing patterns are indeed dynamic, with a significant number of patients experiencing worsening within a year. This deterioration is linked to a progressive decline in quality of life, independent of any structural changes in the pancreas [49].

## 3. Recurrent Acute Pancreatitis: The Critical and Understudied Transition Zone

### 3.1. Why Recurrent Acute Pancreatitis Represents a Distinct Pain Phenotype

The transition from acute to RAP represents a critical but understudied phase in the natural history of pancreatic disease. RAP is defined as two or more episodes of AP with complete or near-complete clinical resolution between attacks, in the absence of established CP, and it is a significant risk factor for progression to CP [50,51]. Although historically treated as a repeated version of AP, accumulating evidence suggests that RAP constitutes a distinct pain phenotype, one shaped not only by the severity of individual episodes but by the nervous system’s memory of what preceded them.

The burden of pain and the impact on quality of life in RAP are considerably more significant than in healthy individuals, although they are notably less severe than in established CP. This gradient indicates the progressive nature of neural remodeling rather than a distinct change between categories [52,53]. Notably, this progression may not be strictly linear at the peripheral level: a recent case–control quantitative sensory testing study comparing patients with distinct pancreatic diseases found that somatosensory deficits in the pancreatic viscerotome were, in several parameters, more pronounced in AP than in established CP [54]. This finding suggests that alterations in pain processing are already detectable early in the disease course, reinforcing the rationale for treating RAP, rather than CP alone, as the critical window for mechanistic study and intervention.

This intermediate phenotype represents a pain system undergoing active transformation, where central changes are detectable but have not yet consolidated into the persistent, self-sustaining sensitization characteristic of established CP. In CP stage, central sensitization increasingly dominates and becomes resistant to treatment. Longitudinal quantitative sensory testing data from a three-year prospective Danish cohort study, which included 35 patients with RAP, reveal that central pain processing phenotypes are not static: within the first year, 46% of patients experienced a change in their sensitization phenotype, with approximately equal numbers showing either deterioration or improvement [49]. This dynamism distinguishes RAP from the more fixed sensitization profile of established CP and suggests that central engagement during the RAP stage retains a degree of plasticity with direct therapeutic implications.

Biologically, RAP holds an important yet often underappreciated position in the pancreatitis continuum. It is neither the isolated inflammatory event of first-episode AP nor the established neuropathic state of CP. It represents the phase during which the nervous system experiences gradual, potentially reversible changes. The Sentinel AP Event (SAPE) hypothesis, proposed by Whitcomb and colleagues, presents the initial episode of AP as an immunological priming event that fundamentally alters the pancreatic microenvironment by sensitizing resident immune cells, lowering the threshold for inflammatory recurrence, and creating a substrate upon which each subsequent episode causes disproportionate injury [55].

The genetic framework further highlights the distinct nature of RAP as a phenotype. Genetic variants in PRSS1 (cationic trypsinogen), SPINK1 (serine protease inhibitor Kazal type 1), CFTR (cystic fibrosis transmembrane conductance regulator), and CTRC (chymotrypsin C) are consistently found in higher proportions within RAP populations. These variants interfere with the protease-antiprotease balance in the acinar cell, decreasing the threshold for premature activation of trypsinogen, thereby increasing the risk of repeated inflammatory episodes [56]. Importantly, these variants not only enhance the likelihood of recurrence but also establish a pro-inflammatory environment in the acini. With each episode, this environment progressively exposes pancreatic nociceptors to elevated cumulative amounts of trypsin, bradykinin, and proteolytic mediators that can directly activate and sensitize TRPV1-expressing afferent neurons [17]. The genetic substrate of RAP is therefore not only a risk factor for recurrence but a molecular amplifier of peripheral sensitization.

Taken together, the clinical dissociation between inflammation and pain, the SAPE-defined neural priming framework, and the genetically determined acinar vulnerability collectively justify treating RAP as a distinct pain phenotype. This distinction necessitates specific mechanistic investigation instead of relying on models from either AP or CP. Preclinical modeling is beginning to reflect this shift: a recent study directly comparing acute and recurrent acute cerulein-induced pancreatitis across mouse strains demonstrated that recurrent, but not single, episodes produce strain-dependent differences in trypsin activity and tissue injury, underscoring that RAP behaves as a mechanistically distinct experimental entity rather than a simple repetition of AP [57].

### 3.2. Incomplete Resolution Between Episodes: Cumulative Peripheral Sensitization

As outlined in Section 2, the neuroimmune alterations that occur during an initial episode of AP such as perineural inflammation, mast cell recruitment, and the increased activity of nociceptive ion channels, do not completely revert to their original state once clinical symptoms subside. In the case of RAP, this partial resolution is not an incidental finding. It serves as the fundamental process through which each subsequent episode exacerbates the already sensitized condition left by the previous one.

At the peripheral level, the gradual increase in sensitivity in RAP is caused by the continuous upregulation of TRPV1 and TRPA1 in pancreatic afferents during repeated inflammatory events. In a rodent model of AP using caerulein, Schwartz and colleagues found that even a single episode leads to a prolonged rise in the expression and function of TRPV1 and TRPA1 in pancreatic DRG and nodose ganglion neurons, which persists beyond the morphological resolution of inflammation [17]. In a later RAP model, in which caerulein was administered twice weekly for up to ten weeks, the same researchers demonstrated that this sensitization does not reset between episodes. Each subsequent episode encounters an afferent population already operating at elevated excitability, promoting neurogenic inflammation and increased sprouting of CGRP-immunoreactive sensory nerve fibers within the pancreatic tissue [58]. This behavioral and electrophysiological signature of cumulative peripheral sensitization has since found some support using a shorter, more concentrated repeated-dosing paradigm. Repeated cerulein administration over two days produced measurable mechanical hypersensitivity in mice, alongside increased excitability of thoracic dorsal root ganglion neurons. Notably, this effect was also reproduced in cerulein-treated human dorsal root ganglion neurons, offering a rare direct preclinical-to-human bridge for this mechanism [59]. The involvement of NGF and TrkA signaling in this context has not been directly explored in RAP models. Information regarding the NGF–TRPV1 pathway in pancreatic pain is solely derived from CP studies and cannot be applied to RAP without further specific research. Importantly, the increase in TRPV1 observed in RAP does not revert to normal after the acute inflammation subsides; instead, it remains between episodes, serving as a molecular basis that ensures each new episode encounters a more sensitive peripheral nociceptive system than the previous one.

Although the upregulation of TRPV1 and TRPA1 is recognized, the specific molecular pathways that lead to cumulative peripheral sensitization during repeated RAP episodes remain largely unexplored. Existing knowledge about mast cell-PAR-2 signaling, the neural communication between the duodenum and pancreas, and calcium-driven changes in DRG is derived solely from acute or CP models, with no research conducted in the context of recurrence.

## 4. Chronic Pancreatitis: From Peripheral Sensitization to Central Neuropathic Pain

### 4.1. Spinal Cord Sensitization: Microglial Activation and NMDA Receptor-Mediated Hyperexcitability

CP is characterized by progressive fibrosis, acinar cell loss, ductal changes, and persistent or recurrent pain in the majority of patients. Pain in CP cannot be explained only by peripheral nociceptive model. Increasing experimental and clinical evidence suggests that as the disease progresses, the central nervous system experiences ongoing neuroplastic changes [60]. The continuous influx of signals from the persistently inflamed pancreatic tissue triggers a series of alterations in the thoracic dorsal horn. Over time, this area shifts from being a regulated somato-visceral relay center to becoming an independent neuroinflammatory enhancer. This transformation involves the hyperphosphorylation of NMDA receptor subunits, activation of microglia via the p38 MAPK pathway, and a gradual breakdown of descending inhibitory control, which is evident as a reduction in conditioned pain modulation (CPM/DNIC) [60].

In CP, central sensitization is clinically evident through symptoms such as allodynia, extensive hyperalgesia that reaches far beyond the pancreatic dermatome (T10), and the enlargement of areas experiencing referred pain, characteristics that cannot be fully accounted for by local organ pathology alone. In a study by Knoph et al., involving a multimodal QST paradigm with 17 CP patients and 20 matched controls, a 45% decrease in pressure pain detection thresholds was observed. Additionally, there was a reduction in cold pressor endurance time and, importantly, an increase in the temporal summation of repeated pain stimuli. Temporal summation, the psychophysical correlate of wind-up, directly indicates increased synaptic efficacy in dorsal horn neurons [61]. To explore spinal excitability more thoroughly, the study utilized the nociceptive withdrawal reflex (NWR), a polysynaptic spinal reflex whose threshold serves as a recognized indicator of dorsal horn excitability. The findings showed that patients with CP had considerably reduced NWR thresholds and increased NWR magnitude in comparison to control subjects, offering the first direct neurophysiological evidence of increased spinal cord excitability in individuals with CP [61]. These findings extend earlier observations by Dimcevski et al., who reported significantly enlarged referred pain areas to electrical stimulation of the esophagus, stomach, and duodenum in CP patients compared to controls, consistent with central receptive field expansion [62]. Additionally, Bouwense et al. demonstrated that the severity of generalized hyperalgesia correlated with disease stage. Patients exhibiting a constant pain pattern showed the most pronounced sensory abnormalities across multiple dermatomes [63].

At the synaptic level, the fundamental mechanism behind central sensitization is the disruption of the Mg^2+^ blockade and the prolonged activation of NMDA receptors in dorsal horn neurons. Normally, these receptors are maintained in a dormant state due to voltage-dependent Mg^2+^ blockage and only activate when the membrane depolarization reaches a certain threshold. With ongoing nociceptive stimulation, co-released neuromodulators, such as substance P, CGRP, and BDNF, all of which show increased levels in experimental chronic pain, gradually reduce the depolarization threshold, allowing for NMDA receptor activation, calcium entry, and subsequent kinase cascades that sustain and enhance synaptic efficacy [60,64]. In a blinded crossover trial, the clinical importance of this mechanism in CP was demonstrated. The study involved administering intravenous S-ketamine (2 µg·kg^−1^·min^−1^ over 3 h), a non-competitive NMDA antagonist, which significantly elevated pressure pain thresholds across several dermatomes, including those far from T10, in 10 CP patients. This offers direct human evidence that NMDA-mediated spinal hyperexcitability in CP is not limited to specific segments but is instead generalized [65]. At the molecular level, TNBS-induced CP in rats is associated with significant phosphorylation of the NR1 and NR2B subunits of the NMDA receptor (pNR1 and pNR2B) in the thoracic dorsal horn. Intrathecal resolvin D1 (RvD1, 100 ng/kg), a pro-resolving lipid mediator, reversed both mechanical allodynia and the phosphorylation of both subunits. Immunofluorescence co-labeling demonstrated that NR1 and NR2B co-localize not only with the neuronal marker NeuN but also with the astrocytic marker GFAP and the microglial marker OX42. This indicates that NMDA receptor-mediated excitatory tone is embedded within a glioneuronal network and is not confined to neurons alone [66].

Microglia play a fundamental role in spinal hyperexcitability, acting as a causal factor rather than just responding to it. In the TNBS rat model of CP, Liu et al. found that microglia in the thoracic dorsal horn take on an activated ameboid shape, with significantly increased levels of phosphorylated p38 MAPK (P-p38) that are exclusively found in OX42-positive cells [67]. Intrathecal minocycline, known for its selective inhibition of microglia, effectively both prevented and reversed the rise in nocifensive behaviors and P-p38 levels, demonstrating a causal role rather than just a correlational one. Importantly, intrathecal fractalkine (CX3CL1), a neuronal chemokine that is consistently expressed and communicates with microglia through its unique receptor CX3CR1, triggered new visceral hyperalgesia in previously unaffected animals. This effect was completely inhibited by minocycline, highlighting the CX3CL1/CX3CR1 pathway as a molecular link between the ongoing primary afferent activity and the recruitment of microglia in the dorsal horn [67]. When activated through p38 MAPK, microglia secrete IL-1β, TNF-α, and IL-6, which enhance synaptic responses to glutamate, inhibit GABAergic and glycinergic interneurons, and promote the unblocking of NMDA receptors. These complete an excitatory loop between glial and neuronal cells [66,67].

In addition to the role of microglia, the expression of Toll-like receptor 3 (TLR3) on astrocytes in the thoracic dorsal horn significantly increases after CP induction in the TNBS model. Immunofluorescence double-labeling reveals that TLR3 is mainly found on GFAP-positive astrocytes, with little presence on microglia and none on neurons. This indicates that astrocytes contribute to spinal neuroinflammatory amplification through a separate, molecularly unique pathway of innate immune sensing [68].

These microglial and astrocytic mechanisms have been characterized exclusively in rodent models of CP [67,68]. Because they require direct spinal cord sampling, they cannot currently be studied in human subjects, representing the most structurally limited pathway in terms of translational validation among those discussed in this review (Section 5).

### 4.2. Descending Modulation and Supraspinal Reorganization: DNIC/CPM Dysfunction, vlPAG Dysfunction, ACC Glutamate Elevation and Descending Facilitation

Overlaying this spinal hyperexcitability is a gradual breakdown of descending inhibitory control, measurable through the Diffuse Noxious Inhibition Control (DNIC) paradigm, known as Conditioned Pain Modulation (CPM) in human studies. In a controlled study involving 25 CP patients and 15 healthy volunteers, Olesen et al. identified three concurrent abnormalities: a marked reduction in DNIC effectiveness, increased sensitivity to electrical and thermal stimulation of the rectosigmoid, and enhanced response to cold pressor tests. This study provides the first significant clinical evidence of compromised descending inhibition in CP [69].

CPM was observed to have an inverse relationship with both the severity of pain and the duration of the disease in larger cohorts undergoing QST. This observation brings forth the unresolved yet clinically significant question of whether dysfunction in the DNIC is a result of persistent nociceptive stimulation, a predisposition to the chronicity of pain, or possibly a combination of both [61,70].

A rodent CP model further suggested that descending facilitatory processes originate in the rostral ventromedial medulla (RVM), accompanied by an increase in spinal dynorphin levels. This peptide, at physiological concentrations, unexpectedly enhances rather than suppresses pain transmission through excitatory mechanisms that do not involve κ-opioid receptors [60]. The transition from descending inhibition to facilitation likely explains the well-known ineffectiveness of opioid-based pain relief in numerous CP patients and justifies the use of centrally acting painkillers. In a randomized, double-blind, placebo-controlled study involving 64 CP patients, pregabalin, a ligand targeting the α2δ subunit of voltage-gated calcium channels that decreases presynaptic glutamate release, significantly alleviated pain compared to a placebo. This effect was particularly notable in patients exhibiting segmental hyperalgesia at T10, a phenotypic indicator of active spinal sensitization [71]. Importantly, pregabalin increased global pain thresholds without restoring CPM/DNIC function [72], indicating that spinal sensitization and descending inhibitory dysfunction are mechanistically dissociable and may require distinct, separately targeted therapeutic strategies. This concept is now embedded in current international consensus recommendations for pain assessment and management in CP [70,73].

Chronic pancreatic pain leads to ongoing molecular restructuring at the supraspinal level, changing the dynamic from descending inhibition to persistent facilitation of nociception. The anatomical substrate of this descending failure lies principally in the vlPAG, which is the main origin of descending antinociceptive signals through the rostral ventromedial medulla, and shows a decrease in presynaptic glutamate release and a reduction in postsynaptic responses mediated by AMPA receptors in experimental CP. In their study using whole-cell patch-clamp recordings on a DBTC-induced rat model, Liu et al. found that vlPAG neurons exhibit smaller excitatory postsynaptic currents, an increased paired-pulse ratio, and decreased AMPA receptor-mediated currents when compared to control subjects. Additionally, the intra-vlPAG microinjection of AMPA was shown to reduce abdominal mechanical hypersensitivity, supporting the idea that weakened glutamatergic synaptic strength in the vlPAG plays a role in sustaining visceral pain. [74].

At the cortical level, the ACC plays a critical role in amplifying both the sensory and emotional aspects of pain. Through anterograde tracing and immunostaining in a rat model of chronic pain induced by TNBS, Ren et al. discovered a direct nociceptive pathway from the NTS to the ACC and demonstrated that chronic pain progressively increases VGluT1 expression while boosting the membrane trafficking and phosphorylation of GluR1 and NR2B through the AC1–cAMP–PKA signaling pathway. This process aligns with long-term potentiation of excitatory synaptic transmission [75]. Clinically, Hansen et al. confirmed translational relevance using MR spectroscopy: CP patients showed significantly elevated glutamate/creatinine ratios in the ACC compared to healthy controls, with the degree of elevation correlating positively with reported pain severity [76]. The lateral parabrachial nucleus (LPB) enhances the transmission of pain signals by promoting NR2B upregulation and the release of glutamate in response to CP. Silencing the glutamatergic neurons in the LPB using chemogenetics notably reduced mechanical hypersensitivity in a caerulein-induced CP model [77]. These findings collectively outline a convergent supraspinal phenotype in CP, characterized by the failure of vlPAG inhibition, amplifying the transmission through synapses in the ACC, and the intensification of ascending signals driven by the LPB—a phenotype mechanistically continuous with, rather than separate from, the DNIC/CPM deficits described above. It is crucial to recognize that the experimental models utilized, DBTC, TNBS, and caerulein, do not entirely replicate the genetic variability and progression of the disease as observed in human CP, necessitating caution when considering direct translational applications.

### 4.3. Neural Remodeling: Nerve Hypertrophy, NGF/TrkA, Artemin and Neuropathic Sprouting

Intrapancreatic neural remodeling is characterized by increased nerve density, fiber hypertrophy, and aberrant axonal sprouting. This remodeling forms a structural basis for the chronic pain experienced in CP, which persists independently of acute inflammatory activity. In an extensive pathomorphological investigation involving 546 patients, Ceyhan et al. demonstrated that both neural hypertrophy and elevated nerve density are nearly universal observations in CP. These findings are directly linked to the intensity of pain and establish pancreatic neuropathy as a primary contributor to the pain phenotype rather than a secondary one [78].

The principal molecular driver of this remodeling is the NGF/TrkA axis. In CP, the expression of NGF expands beyond its typical islet-specific distribution to include metaplastic ductal cells and deteriorating acini. When NGF binds to TrkA, it triggers PI3K/Ras signaling in pain-sensitive neurons, reducing activation thresholds and encouraging axonal growth. Notably, NGF plays a crucial role in the upregulation and sensitization of TRPV1 in pancreatic sensory neurons. In a CP rat model induced by TNBS, the application of an anti-NGF antibody to neutralize NGF effectively reversed pancreatic hyperalgesia and led to a marked reduction in both TRPV1 current density and TRPV1 expression in neurons of the dorsal root ganglia, thereby establishing a direct connection between the NGF/TrkA/TRPV1 pathway and the clinical presentation of pain [79].

The binding of NGF to TrkA initiates PI3K/Ras signaling in nociceptive neurons, which decreases activation thresholds, increases the expression of TRPV1 and Nav1.8, and encourages axonal sprouting. Recent research has bolstered the clinical significance of this pathway: the use of anti-NGF antibodies for systemic NGF sequestration in a genetically modified mouse model resulted in a notable decrease in nociceptive behaviors and restored TrkA and p75 mRNA levels in thoracic dorsal root ganglia, thereby validating NGF/TrkA as a viable therapeutic target for pancreatic neuropathy [80].

Moreover, artemin, a member of the GDNF family, serves as a secondary neurotrophic signal that promotes neuropathic sprouting by interacting with the GFRα3–RET receptor complex. In 66 CP samples, there was a marked overexpression of both artemin and GFRα3, which were found in Schwann cells, neural ganglia, and arterial smooth muscle. The levels of artemin mRNA were linked to the severity of pain, the extent of perineural inflammatory infiltration, neural density, and hypertrophy. Importantly, activated pancreatic stellate cells increase artemin expression in response to TGF-β1, thereby molecularly linking ongoing fibrogenesis with neuropathic axonal sprouting [81].

Together, these findings position the NGF/TrkA-artemin axis as a central driver of neural remodeling in CP. While the causal, interventional evidence for this pathway derives from rodent models [79,80], the artemin data above are drawn directly from human CP tissue [81], illustrating the mixed evidentiary basis, animal mechanistic and human correlative, that characterizes much of this field. The implications of this distinction are discussed in Section 5.

### 4.4. Mast Cell–Nerve Crosstalk, Schwann Cell Activation and Perineurial Damage

Beyond neural remodeling, the perineural microenvironment in CP is actively shaped by immune-neural interactions that sustain and amplify nociception. Two cellular players are central to this process: mast cells and Schwann cells.

In cases of painful CP, mast cells tend to gather predominantly around intrapancreatic nerves. A quantitative immunolabeling study conducted by Demir et al. on 20 CP samples revealed that mast cells constitute about 27% of all perineural inflammatory cells. These cells are notably more prevalent around nerves in patients experiencing pain compared to those without pain [82]. Sensory nerve fibers lie close to mast cells, forming a self-amplifying neuro-immune loop. Activated afferents release neuropeptides—mainly substance P, CGRP, and NGF—that bind to mast cell receptors and trigger degranulation. The released tryptase activates PAR-2, a receptor on most pancreatic nociceptive neurons. Hoogerwerf et al. showed that trypsin activates PAR-2, which then activates dorsal horn neurons and causes pain in rats. This links mast cell degranulation directly to pancreatic pain [83].

Activation of PAR-2 further enhances the sensitivity of TRPV1, leading to increased antidromic neuropeptide release and completing the cycle. The involvement of mast cells was validated in mast cell-deficient (Kit^W^/Kit^W-v^) mice, where their absence notably reduced pain behavior in experimental CP [84].

Schwann cells, the glial cells surrounding peripheral nerves, adopt a reactive phenotype in CP by upregulating the chemokine CX3CL1 and its receptor CX3CR1. Ceyhan et al. found that both are overexpressed in CP, especially in patients with severe pain and active pancreatic neuritis. CX3CR1 expression significantly correlates with nerve density, hypertrophy, and severity of fibrosis [85]. Reactive Schwann cells secrete fractalkine, recruiting macrophages and cytotoxic T lymphocytes into the perineural space, which amplifies neuritis and disrupts the perineurial barrier. Once this barrier is breached, proteases and cytokines can access axons and Schwann cells, leading to structural nerve damage and nociceptive sensitization, making the intrapancreatic nerve a source of pain [86].

## 5. Species-Specific Limitations of Experimental Pain Models

Throughout this review, the species, tissue source, or evidentiary basis, human, animal, in vitro, or extrapolated, of each cited finding has been stated explicitly, in order to distinguish preclinical from clinical evidence rather than conflate the two. This distinction is addressed in detail below.

Much of the mechanistic understanding of pain in pancreatitis presented in this review derives from rodent and, to a lesser extent, canine models, reflecting the practical and ethical constraints of studying nociceptive signaling directly in human tissue. While these models have been indispensable for identifying candidate pathways, their translational validity varies considerably depending on the mechanism in question and should be interpreted with corresponding caution.

The trypsin-PAR2-TRPV1 signaling cascade, central to current models of peripheral sensitization in AP, has been characterized almost exclusively in mouse and rat models [10,11,12,13,14,15,16,17,18,20,83] using intraductal trypsin or PAR2-activating peptide administration combined with behavioral nociceptive assays. Direct evidence for this pathway’s activity in human pancreatic tissue during pancreatitis is currently lacking; the closest available human data derive from pancreatic cancer tissue, where PAR2 colocalizes with neuronal markers and sensitizes dorsal root ganglion neurons [87], a related but pathophysiologically distinct context. Extrapolation of this pathway’s role in human pancreatitis pain therefore remains inferential.

RAP occupies a comparatively more favorable position on the translational spectrum than the AP-specific pathway discussed above. Cumulative peripheral sensitization through repeated TRPV1/TRPA1 upregulation, originally characterized in repeated-caerulein rat models [58], has recently found partial support in a paradigm directly bridging mouse and human tissue: repeated cerulein exposure produced measurable mechanical hypersensitivity in mice alongside increased excitability in both murine and human dorsal root ganglion neurons [59]. This represents one of the few instances in this review where a peripheral sensitization mechanism has been demonstrated directly in human sensory neurons, rather than inferred from rodent data alone. At the systems level, human evidence is further supported by quantitative sensory testing studies showing measurable, dynamic alterations in pain processing in RAP patients [49], and by findings that somatosensory changes may already be detectable in the acute setting [54]. Preclinical modeling of RAP as a mechanistically distinct entity, rather than repeated AP, is also emerging [57], although this model characterizes tissue injury and enzymatic activity rather than nociceptive behavior directly. As the following two mechanisms illustrate, however, this more favorable translational picture does not extend uniformly to CP.

The NGF/TrkA pathway occupies an intermediate position. Rodent models have established that NGF upregulation sensitizes pancreatic nociceptors and that systemic anti-NGF administration reduces pancreatic hyperalgesia [29,30,64,79,80] but such functional interventional evidence cannot ethically be replicated in human subjects. Nonetheless, correlative human data exist: TrkA expression in human CP tissue has been shown to correlate with reported pain severity [88], offering partial support for the relevance of this pathway beyond the animal model. This illustrates a broader point: for several pathways, human evidence, where available, tends to be correlative and tissue-based rather than functional or interventional, and should not be conflated with mechanistic confirmation.

The spinal microglia-p38-NMDA pathway represents the pathway with the least direct translatability. Because it requires access to spinal cord tissue, this mechanism has been characterized exclusively in rat models of CP [6,67,68,77]. No direct human correlate is feasible using current methodology; the closest available human evidence comes from quantitative sensory testing and functional neuroimaging studies [1,49,61,62,63], which demonstrate behavioral and functional signatures consistent with central sensitization but cannot confirm the underlying cellular mechanisms identified in rodents. This represents a structural, rather than a merely temporary, gap in translatability.

Beyond pathway-specific limitations, broader interspecies differences in nociceptor biology, immune signaling, and pain-related behavior further complicate direct extrapolation from rodent findings to human pathophysiology. Rodent studies rely on behavioral proxies for pain (e.g., mechanical withdrawal thresholds, nocifensive responses) that, while validated, are indirect measures compared to self-reported pain in human studies. Consequently, the mechanistic pathways discussed throughout this review should be understood as biologically plausible and, in some cases, partially supported by human correlative data, rather than as fully validated determinants of human pancreatitis pain. This distinction carries direct implications for the interpretation of preclinical therapeutic targets discussed in the following section.

## 6. Therapeutic Implications of the Molecular Framework

Understanding the molecular basis of pain in CP is essential for developing targeted therapies that go beyond symptomatic relief. NGF/TrkA and TRPV1 have been identified as key drivers of peripheral sensitization. Experimental interventions, such as anti-NGF antibodies and TRPV1 antagonists, have successfully reduced pain behavior in animal models of CP, suggesting their potential as analgesic targets [79]. However, the clinical translation of these preclinical findings has been markedly uneven. Anti-NGF antibodies (e.g., tanezumab) demonstrated meaningful analgesic efficacy across multiple Phase 3 trials in osteoarthritis and chronic low back pain, and a dedicated trial in CP patients was in fact initiated [89]. However, this trial was terminated after enrolling only two participants when the U.S. FDA imposed a class-wide clinical hold on NGF-inhibitor development in December 2010 due to reports of osteonecrosis and rapidly progressive osteoarthritis. Despite subsequent risk-mitigation strategies, an FDA advisory committee voted against approval of tanezumab in 2021, and its global clinical development program was discontinued later that year [90]. No efficacy or safety data specific to pancreatitis were therefore ever generated, leaving the translational potential of this target in CP formally untested rather than disproven.

TRPV1 antagonists have similarly shown proof-of-mechanism analgesic effects in early-phase human trials, but clinical development of most compounds was halted due to a consistent class-wide adverse effect—drug-induced hyperthermia, in some cases exceeding 40 °C, rather than lack of efficacy [91]. At the neuro-immune interface, the mast cell–PAR-2–TRPV1 loop represents a tractable point of intervention, as PAR-2 blockade can interrupt the cycle of neuronal sensitization driven by mast cell degranulation [83]. Clinically, however, PAR-2 antagonism remains at a considerably earlier stage, with the first PAR-2-targeted antibody (MEDI0618) having only recently entered a first-in-human Phase 1 safety and tolerability trial, without any pain-specific efficacy data yet available [92]. A comparable situation applies to CX3CR1 blockade: the selective antagonist KAND567 (AZD8797) has entered clinical development, but exclusively for non-pain indications, including COVID-19 and oncology, with safety and pharmacokinetic data available in healthy volunteers [93]. No clinical trial to date has evaluated CX3CR1 blockade for pain in any condition, including pancreatitis.

Recognizing that some CP patients have supraspinal reorganization with increased ACC glutamate and impaired inhibition explains why pancreatic interventions often fail for pain relief, highlighting the need for centrally acting agents [94]. Collectively, this molecular framework argues for a mechanism-based, patient-stratified approach to CP pain management, moving away from a one-size-fits-all analgesic strategy toward treatments matched to each patient’s dominant pain phenotype. Importantly, clinical translation across these targets sits at markedly different stages: halted for safety reasons despite demonstrated efficacy (anti-NGF, TRPV1), or not yet advanced to pain-specific efficacy testing (PAR-2, CX3CR1).

These divergent clinical trajectories carry direct implications for future therapeutic development in CP pain. First, existing trial data come primarily from osteoarthritis and musculoskeletal pain cohorts; safety findings from these populations (joint safety for anti-NGF, thermoregulatory effects for TRPV1 antagonists) may not directly translate to a visceral, pancreatic pain context with different dosing needs and baseline pathology. Second, the absence of pancreatitis-specific efficacy data means that matching a given target to a patient’s dominant pain phenotype, peripheral, nociceptive-driven pain in early RAP versus established central sensitization in advanced CP, remains a theoretical rather than evidence-based strategy. Prospective trials using QST-defined sensory phenotypes as an enrollment criterion would be needed to test this stratification approach directly. Until such data exist, the therapeutic framework outlined above should be considered mechanistically plausible but clinically unproven.

Beyond characterizing the molecular mechanisms of pain, multi-omic profiling reveals a landscape of unaddressed therapeutic targets whose relevance shifts across the pancreatic pain continuum, from genotype-driven nociceptor sensitization in early disease to transcriptomic markers of neural remodeling and metabolic drivers of ischemic pain in advanced stages (Table 1).

## 7. Conclusions

Pain in pancreatitis is the result of a complex, multidimensional process involving peripheral sensitization, structural neural remodeling, and progressive central reorganization. The role each mechanism plays varies among individuals and at different stages of the disease, which is why a single treatment approach is not universally successful. Significantly, the persistence of pain in certain patients even after undergoing a total pancreatectomy indicates that, in advanced stages, pain may become completely independent of the pancreas and be driven solely by alterations in the central nervous system. These findings strongly support a personalized, mechanism-driven strategy for managing pain in pancreatitis, taking into account not just the condition of the pancreas but also the unique neurobiological characteristics of each individual.

## Figures and Tables

**Table 1:** Multi-omic landscape of pancreatic pain: molecular mechanisms, phase relevance, and emerging therapeutic targets.

Omic Level	Molecular Biomarker/Target	Evidence Level	Cellular/Tissue Source	Specific Role in Pain Chronification	Future Therapeutic Target/Strategy	AP	RAP	CP
Genomics	*TRPV1* variants (e.g., rs222747)	EX	Germline DNA	rs222747 increases TRPV1 protein expression at cell surface; potentially lowers nociceptor activation threshold and predisposes to more pronounced acute pain responses. Association with CP not established in human cohorts.	Genotype-guided TRPV1 antagonists (capsazepine analogues); personalised analgesic dosing based on rs222747 carrier status. (Preclinical rationale; no clinical trials in pancreatitis to date.)	●	○	○
Genomics	*PRSS1*/*SPINK1* mutations	HV	Acinar cells	Impaired trypsinogen autoactivation control → sustained intracellular and luminal trypsin activity → trypsin-mediated PAR-2 activation on afferent fibers → persistent nociceptive drive and progressive nerve remodeling.	PAR-2 antagonists (GB88 analogues) in genotype-selected patients; early endoscopic or surgical decompression before irreversible neural remodeling is established.	○	●	●
Transcriptomics	*GAP43* mRNA (Growth Associated Protein 43)	HT	Intrapancreatic nerves	Marker of active axonal sprouting and neural plasticity; upregulated in hypertrophic intrapancreatic nerves in CP; GAP43+ fiber density correlates with pain intensity and dorsal horn activation.	Anti-NGF antibodies (e.g., tanezumab) to interrupt the sprouting signal upstream of GAP43 upregulation; GAP43 mRNA as liquid biopsy target. (Preclinical rationale only; not yet evaluated in pancreatitis clinical trials.)	○	●	●
Transcriptomics	*CX3CL1* (Fractalkine) mRNA	HT	Endothelial cells/intrapancreatic neurons	Neural and endothelial CX3CL1 recruits CX3CR1+ macrophages and lymphocytes to form perineural insulitis; neural CX3CL1 immunoreactivity correlates with pancreatic neuritis severity, nerve fiber hypertrophy, and pain intensity in CP.	Anti-CX3CL1 neutralising antibodies or CX3CR1 antagonists (AZD8797) to prevent macrophage-driven perineural invasion and attenuate neuropathic pain progression in CP.	○	●	●
Metabolomics	Elevated Succinate & Lactate	EX	Ischemic/Hypoxic acinar tissue	Succinate activates SUCNR1 on nociceptors triggering PLCβ-mediated Ca^2+^ mobilisation and neuronal hypersensitivity; lactate lowers local pH activating ASICs on afferent fibers. Mechanism inferred from pain neuroscience; direct validation in pancreatic nociception pending.	ASIC3 blockers (APETx2 analogues); SUCNR1 antagonists to interrupt metabolic nociceptive signaling. (Extrapolated from general pain models; pancreatic-specific trials lacking.)	●	●	●
Metabolomics	Lipidomics: 2-AG & AEA (Endocannabinoids)	PC	Pancreatic parenchyma/Inflammatory cells	In AP, CB1/CB2 receptors and endocannabinoid levels are upregulated as a partially protective anti-nociceptive response. In RAP/CP, progressive degradation of local endocannabinoid tone (via FAAH/MAGL) removes CB1/CB2-mediated inhibition, promoting afferent sensitisation and central pain amplification.	FAAH inhibitors (URB597) or MAGL inhibitors (JZL184) to restore 2-AG/AEA tone in RAP/CP; peripheral CB2 agonists to reduce neuroinflammation without CNS effects. (Preclinical evidence in pancreatitis models; clinical trials not yet conducted.)	●	●	●

Abbreviations: 2-AG, 2-arachidonoylglycerol; AEA, anandamide; AP, acute pancreatitis; ASIC, acid-sensing ion channel; CB1/CB2, cannabinoid receptors 1 and 2; CP, chronic pancreatitis; CX3CL1/CX3CR1, fractalkine ligand and receptor; EX = Extrapolated (evidence from non-pancreatic pain models or general human genetics, not yet validated in pancreatitis), EUS-FNA, endoscopic ultrasound-guided fine needle aspiration; FAAH, fatty acid amide hydrolase; GAP43, growth-associated protein 43; HV = Human, validated (established human genetic/clinical association); HT = Human tissue, correlative (derived from human tissue studies, non-interventional); MAGL, monoacylglycerol lipase; NGF, nerve growth factor; PAR-2, protease-activated receptor 2; PC = Preclinical, pancreatitis-specific (evidence from pancreatitis animal/cell models); PLCβ, phospholipase Cβ; PRSS1, cationic trypsinogen gene; RAP, recurrent acute pancreatitis; SPINK1, serine protease inhibitor Kazal type 1; SUCNR1, succinate receptor 1; TRPV1, transient receptor potential vanilloid 1. **●** predominant phase; ○ not predominant. → indicates a sequential/causal progression between mechanistic steps (i.e., leads to).

## Data Availability

No new data were created or analyzed in this study. Data sharing is not applicable to this article.

## References

[1] Drewes A.M., Gratkowski M., Sami S.A., Dimcevski G., Funch-Jensen P., Arendt-Nielsen L. (2008). Is the Pain in Chronic Pancreatitis of Neuropathic Origin? Support from EEG Studies during Experimental Pain. World J. Gastroenterol..

[2] Ghorbani P., Dankha R., Brisson R., D’Souza M.A., Löhr J.-M., Sparrelid E., Vujasinovic M. (2022). Surgical Outcomes and Trends for Chronic Pancreatitis: An Observational Cohort Study from a High-Volume Centre. J. Clin. Med..

[3] Lin Z., Pandol S., Apte M., Jiang Y. (2025). Navigating Chronic Pancreatitis Pain: A Pathophysiological and Therapeutic Overview. Front. Physiol..

[4] Demir I.E., Tieftrunk E., Maak M., Friess H., Ceyhan G.O. (2011). Pain Mechanisms in Chronic Pancreatitis: Of a Master and His Fire. Langenbecks Arch. Surg..

[5] Poulsen J.L. (2013). Pain and Chronic Pancreatitis: A Complex Interplay of Multiple Mechanisms. World J. Gastroenterol..

[6] Liao Y., Wang J., Wei Y., Zhang T., Zhang Y., Zuo Z., Teng X., Li Y. (2017). Histone Deacetylase 2 Is Involved in µ-opioid Receptor Suppression in the Spinal Dorsal Horn in a Rat Model of Chronic Pancreatitis Pain. Mol. Med. Rep..

[7] Meints S.M., Edwards R.R. (2018). Evaluating Psychosocial Contributions to Chronic Pain Outcomes. Prog. Neuro-Psychopharmacol. Biol. Psychiatry.

[8] Bhatia M., Brady M., Shokuhi S., Christmas S., Neoptolemos J.P., Slavin J. (2000). Inflammatory Mediators in Acute Pancreatitis. J. Pathol..

[9] Nicoletti A., Vitale F., Paratore M., Quero G., Negri M., Nista E.C., Alfieri S., Gasbarrini A., Zileri Dal Verme L. (2024). Neuropancreatology: The Nervous System and Pain Management in Pancreatic Diseases. Life.

[10] Hoogerwerf W.A., Zou L., Shenoy M., Sun D., Micci M.A., Lee-Hellmich H., Xiao S.Y., Winston J.H., Pasricha P.J. (2001). The Proteinase-Activated Receptor 2 Is Involved in Nociception. J. Neurosci..

[11] Ceppa E.P., Lyo V., Grady E.F., Knecht W., Grahn S., Peterson A., Bunnett N.W., Kirkwood K.S., Cattaruzza F. (2011). Serine Proteases Mediate Inflammatory Pain in Acute Pancreatitis. Am. J. Physiol.-Gastrointest. Liver Physiol..

[12] Amadesi S., Cottrell G.S., Divino L., Chapman K., Grady E.F., Bautista F., Karanjia R., Barajas-Lopez C., Vanner S., Vergnolle N. (2006). Protease-activated Receptor 2 Sensitizes TRPV1 by Protein Kinase Cɛ- and A-dependent Mechanisms in Rats and Mice. J. Physiol..

[13] Dai Y., Wang S., Tominaga M., Yamamoto S., Fukuoka T., Higashi T., Kobayashi K., Obata K., Yamanaka H., Noguchi K. (2007). Sensitization of TRPA1 by PAR2 Contributes to the Sensation of Inflammatory Pain. J. Clin. Investig..

[14] Kawabata A., Matsunami M., Tsutsumi M., Ishiki T., Fukushima O., Sekiguchi F., Kawao N., Minami T., Kanke T., Saito N. (2006). Suppression of Pancreatitis-related Allodynia/Hyperalgesia by Proteinase-activated Receptor-2 in Mice. Br. J. Pharmacol..

[15] Wick E.C., Hoge S.G., Grahn S.W., Kim E., Divino L.A., Grady E.F., Bunnett N.W., Kirkwood K.S. (2006). Transient Receptor Potential Vanilloid 1, Calcitonin Gene-Related Peptide, and Substance P Mediate Nociception in Acute Pancreatitis. Am. J. Physiol.-Gastrointest. Liver Physiol..

[16] Nathan J.D., Patel A.A., McVey D.C., Thomas J.E., Prpic V., Vigna S.R., Liddle R.A. (2001). Capsaicin Vanilloid Receptor-1 Mediates Substance P Release in Experimental Pancreatitis. Am. J. Physiol.-Gastrointest. Liver Physiol..

[17] Schwartz E.S., Christianson J.A., Chen X., La J., Davis B.M., Albers K.M., Gebhart G.F. (2011). Synergistic Role of TRPV1 and TRPA1 in Pancreatic Pain and Inflammation. Gastroenterology.

[18] Zhao P., Lieu T., Barlow N., Sostegni S., Haerteis S., Korbmacher C., Liedtke W., Jimenez-Vargas N.N., Vanner S.J., Bunnett N.W. (2015). Neutrophil Elastase Activates Protease-Activated Receptor-2 (PAR2) and Transient Receptor Potential Vanilloid 4 (TRPV4) to Cause Inflammation and Pain. J. Biol. Chem..

[19] Noble M.D., Romac J., Vigna S.R., Liddle R.A. (2008). A pH-Sensitive, Neurogenic Pathway Mediates Disease Severity in a Model of Post-ERCP Pancreatitis. Gut.

[20] Griesbacher T., Rainer I., Tiran B., Peskar B.A. (2008). Kallikrein Inhibitors Limit Kinin B_2_ Antagonist-induced Progression of Oedematous to Haemorrhagic Pancreatitis in Rats. Br. J. Pharmacol..

[21] Pethő G., Reeh P.W. (2012). Sensory and Signaling Mechanisms of Bradykinin, Eicosanoids, Platelet-Activating Factor, and Nitric Oxide in Peripheral Nociceptors. Physiol. Rev..

[22] Choi S.-I., Hwang S.W. (2018). Depolarizing Effectors of Bradykinin Signaling in Nociceptor Excitation in Pain Perception. Biomol. Ther..

[23] Sugiura T., Tominaga M., Katsuya H., Mizumura K. (2002). Bradykinin Lowers the Threshold Temperature for Heat Activation of Vanilloid Receptor 1. J. Neurophysiol..

[24] McLatchie L.M., Fraser N.J., Main M.J., Wise A., Brown J., Thompson N., Solari R., Lee M.G., Foord S.M. (1998). RAMPs Regulate the Transport and Ligand Specificity of the Calcitonin-Receptor-like Receptor. Nature.

[25] Vera-Portocarrero L.P., Westlund K.N. (2004). Attenuation of Nociception in a Model of Acute Pancreatitis by an NK-1 Antagonist. Pharmacol. Biochem. Behav..

[26] Mantyh P.W., DeMaster E., Malhotra A., Ghilardi J.R., Rogers S.D., Mantyh C.R., Liu H., Basbaum A.I., Vigna S.R., Maggio J.E. (1995). Receptor Endocytosis and Dendrite Reshaping in Spinal Neurons After Somatosensory Stimulation. Science.

[27] Le Greves P., Nyberg F., Terenius L., Hökfelt T. (1985). Calcitonin Gene-Related Peptide Is a Potent Inhibitor of Substance P Degradation. Eur. J. Pharmacol..

[28] Wiesenfeld-Hallin Z., Hökfelt T., Lundberg J.M., Forssmann W.G., Reinecke M., Tschopp F.A., Fischer J.A. (1984). Immunoreactive Calcitonin Gene-Related Peptide and Substance P Coexist in Sensory Neurons to the Spinal Cord and Interact in Spinal Behavioral Responses of the Rat. Neurosci. Lett..

[29] Toma H., Winston J., Micci M., Shenoy M., Pasricha P.J. (2000). Nerve Growth Factor Expression Is Up-Regulated in the Rat Model of L-Arginine–Induced Acute Pancreatitis. Gastroenterology.

[30] Winston J.H., Toma H., Shenoy M., He Z.-J., Zou L., Xiao S.-Y., Micci M.-A., Pasricha P.J. (2003). Acute Pancreatitis Results in Referred Mechanical Hypersensitivity and Neuropeptide Up-Regulation That Can Be Suppressed by the Protein Kinase Inhibitor K252a. J. Pain.

[31] Gukovsky I., Gukovskaya A.S., Blinman T.A., Zaninovic V., Pandol S.J. (1998). Early NF-κB Activation Is Associated with Hormone-Induced Pancreatitis. Am. J. Physiol.-Gastrointest. Liver Physiol..

[32] Steinle A.U., Weidenbach H., Wagner M., Adler G., Schmid R.M. (1999). NF-kB/Rel Activation in Cerulein Pancreatitis. Gastroenterology.

[33] Greer P.J., Lee P.J., Paragomi P., Stello K., Phillips A., Hart P., Speake C., Lacy-Hulbert A., Whitcomb D.C., Papachristou G.I. (2022). Severe Acute Pancreatitis Exhibits Distinct Cytokine Signatures and Trajectories in Humans: A Prospective Observational Study. Am. J. Physiol.-Gastrointest. Liver Physiol..

[34] Sternby H., Hartman H., Thorlacius H., Regnér S. (2021). The Initial Course of IL1β, IL-6, IL-8, IL-10, IL-12, IFN-γ and TNF-α with Regard to Severity Grade in Acute Pancreatitis. Biomolecules.

[35] Ji R.-R., Samad T.A., Jin S.-X., Schmoll R., Woolf C.J. (2002). P38 MAPK Activation by NGF in Primary Sensory Neurons after Inflammation Increases TRPV1 Levels and Maintains Heat Hyperalgesia. Neuron.

[36] Vardanyan M., Melemedjian O.K., Price T.J., Ossipov M.H., Lai J., Roberts E., Boos T.L., Deschamps J.R., Jacobson A.E., Rice K.C. (2010). Reversal of Pancreatitis-Induced Pain by an Orally Available, Small Molecule Interleukin-6 Receptor Antagonist. Pain.

[37] Zhou Y.-Q., Liu Z., Liu Z.-H., Chen S.-P., Li M., Shahveranov A., Ye D.-W., Tian Y.-K. (2016). Interleukin-6: An Emerging Regulator of Pathological Pain. J. Neuroinflamm..

[38] Xue M., Han L., Qian W., Li J., Qin T., Xiao Y., Ma Q., Ma J., Shen X. (2020). Nitric Oxide Stimulates Acute Pancreatitis Pain via Activating the NF-*κ*B Signaling Pathway and Inhibiting the Kappa Opioid Receptor. Oxidative Med. Cell. Longev..

[39] Xia C.-C., Chen H.-T., Deng H., Huang Y.-T., Xu G.-Q. (2024). Reactive Oxygen Species and Oxidative Stress in Acute Pancreatitis: Pathogenesis and New Therapeutic Interventions. World J. Gastroenterol..

[40] Armstrong J.A., Cash N.J., Ouyang Y., Morton J.C., Chvanov M., Latawiec D., Awais M., Tepikin A.V., Sutton R., Criddle D.N. (2018). Oxidative Stress Alters Mitochondrial Bioenergetics and Modifies Pancreatic Cell Death Independently of Cyclophilin D, Resulting in an Apoptosis-to-Necrosis Shift. J. Biol. Chem..

[41] Sies H. (2015). Oxidative Stress: A Concept in Redox Biology and Medicine. Redox Biol..

[42] Chuang H., Lin S. (2009). Oxidative Challenges Sensitize the Capsaicin Receptor by Covalent Cysteine Modification. Proc. Natl. Acad. Sci. USA.

[43] Piciu F., Balas M., Badea M., Cucu D. (2023). TRP Channels in Tumoral Processes Mediated by Oxidative Stress and Inflammation. Antioxidants.

[44] Trevisani M., Siemens J., Materazzi S., Bautista D.M., Nassini R., Campi B., Imamachi N., Andrè E., Patacchini R., Cottrell G.S. (2007). 4-Hydroxynonenal, an Endogenous Aldehyde, Causes Pain and Neurogenic Inflammation through Activation of the Irritant Receptor TRPA1. Proc. Natl. Acad. Sci. USA.

[45] Zhang Y., Asgar J., Shou H., Pak J., Da Silva J.T., Ro J.Y. (2023). Intraganglionic Reactive Oxygen Species Mediate Inflammatory Pain and Hyperalgesia through TRPA1 in the Rat. Front. Pain Res..

[46] Bai G., Ross H., Zhang Y., Lee K., Ro J.Y. (2020). The Role of DNA Methylation in Transcriptional Regulation of Pro-Nociceptive Genes in Rat Trigeminal Ganglia. Epigenet. Insights.

[47] Takayama Y., Uta D., Furue H., Tominaga M. (2015). Pain-Enhancing Mechanism through Interaction between TRPV1 and Anoctamin 1 in Sensory Neurons. Proc. Natl. Acad. Sci. USA.

[48] Lee B., Cho H., Jung J., Yang Y.D., Yang D.-J., Oh U. (2014). Anoctamin 1 Contributes to Inflammatory and Nerve-Injury Induced Hypersensitivity. Mol. Pain.

[49] Kuhlmann L., Davidsen L., Knoph C.S., Hadi A., Novovic S., Larsen I.M., Frøkjær J.B., Drewes A.M., Olesen S.S. (2026). Trajectories of Pain Processing in Recurrent Acute and Chronic Pancreatitis: A Longitudinal Quantitative Sensory Testing Study. Eur. J. Pain.

[50] Anderson M.A., Akshintala V., Albers K.M., Amann S.T., Belfer I., Brand R., Chari S., Cote G., Davis B.M., Frulloni L. (2016). Mechanism, Assessment and Management of Pain in Chronic Pancreatitis: Recommendations of a Multidisciplinary Study Group. Pancreatology.

[51] Sankaran S.J., Xiao A.Y., Wu L.M., Windsor J.A., Forsmark C.E., Petrov M.S. (2015). Frequency of Progression From Acute to Chronic Pancreatitis and Risk Factors: A Meta-Analysis. Gastroenterology.

[52] Viswanath A., Rao N.V., Sharma M., Rajesh S., Yugalakshmi A., Sumathy J., Kumbhar G., Thomas A., Kurien R.T., Samanta J. (2026). Quality of Life in Recurrent Acute Pancreatitis and Chronic Pancreatitis: A Prospective Cross-Sectional Comparative Bicentric Study. Indian J. Gastroenterol..

[53] Dunbar E.K., Greer P.J., Amann S.T., Alkaade S., Banks P., Brand R., Conwell D.L., Forsmark C.E., Gardner T.B., Guda N.M. (2021). Pain Experience in Pancreatitis: Strong Association of Genetic Risk Loci for Anxiety and PTSD in Patients With Severe, Constant, and Constant-Severe Pain. Am. J. Gastroenterol..

[54] Göltl P., Merz P., Schneider A., Ebert M.P., Hirth M., Magerl W. (2025). Somatosensory Profiling to Differentiate Distinct Painful Diseases of the Pancreas—A Quantitative Sensory Testing Case-Control Study. Pain.

[55] Whitcomb D.C. (2022). Central Role of the Sentinel Acute Pancreatitis Event (SAPE) Model in Understanding Recurrent Acute Pancreatitis (RAP): Implications for Precision Medicine. Front. Pediatr..

[56] Whitcomb D.C. (2013). Genetic Risk Factors for Pancreatic Disorders. Gastroenterology.

[57] Demcsák A., Tran T., Sahin-Tóth M., Geisz-Fremy A. (2025). Strain-Specific Differences in Cerulein-Induced Acute and Recurrent Acute Murine Pancreatitis. Sci. Rep..

[58] Schwartz E.S., La J.-H., Scheff N.N., Davis B.M., Albers K.M., Gebhart G.F. (2013). TRPV1 and TRPA1 Antagonists Prevent the Transition of Acute to Chronic Inflammation and Pain in Chronic Pancreatitis. J. Neurosci..

[59] Goyal S., Zurek N., Ehsanian R., Goyal S., Jones D.T., Shilling M., Desir G.V., Gorelick F., Westlund K.N., Alles S.R. (2025). Visceral Pain-Related Acute Actions of Cerulein on Mouse and Human Sensory Neurons. Mol. Pain.

[60] Olesen S.S., Krauss T., Demir I.E., Wilder-Smith O.H., Ceyhan G.O., Pasricha P.J., Drewes A.M. (2017). Towards a Neurobiological Understanding of Pain in Chronic Pancreatitis: Mechanisms and Implications for Treatment. Pain Rep..

[61] Knoph C.S., Nedergaard R.B., Olesen S.S., Kuhlmann L., Drewes A.M. (2023). Spinal Excitability in Patients with Painful Chronic Pancreatitis. J. Pain Res..

[62] Dimcevski G., Sami S.A.K., Funch–Jensen P., Le Pera D., Valeriani M., Arendt–Nielsen L., Drewes A.M. (2007). Pain in Chronic Pancreatitis: The Role of Reorganization in the Central Nervous System. Gastroenterology.

[63] Bouwense S.A.W., Olesen S.S., Drewes A.M., Frøkjær J.B., Van Goor H., Wilder-Smith O.H.G. (2013). Is Altered Central Pain Processing Related to Disease Stage in Chronic Pancreatitis Patients with Pain? An Exploratory Study. PLoS ONE.

[64] Hughes M.S., Shenoy M., Liu L., Colak T., Mehta K., Pasricha P.J. (2011). Brain-Derived Neurotrophic Factor Is Upregulated in Rats With Chronic Pancreatitis and Mediates Pain Behavior. Pancreas.

[65] Bouwense S.A.W., Buscher H.C.J.L., Van Goor H., Wilder-Smith O.H.G. (2011). S-Ketamine Modulates Hyperalgesia in Patients With Chronic Pancreatitis Pain. Reg. Anesth. Pain Med..

[66] Quan-Xin F., Fan F., Xiang-Ying F., Shu-Jun L., Shi-Qi W., Zhao-Xu L., Xu-Jie Z., Qing-Chuan Z., Wei W. (2012). Resolvin D1 Reverses Chronic Pancreatitis-Induced Mechanical Allodynia, Phosphorylation of NMDA Receptors, and Cytokines Expression in the Thoracic Spinal Dorsal Horn. BMC Gastroenterol..

[67] Liu P., Lu C., Wang C., Lee I., Hsieh J., Chen C., Lee H., Lin H., Chang F., Lee S. (2012). Spinal Microglia Initiate and Maintain Hyperalgesia in a Rat Model of Chronic Pancreatitis. Gastroenterology.

[68] Qian N.-S., Liao Y.-H., Feng Q.-X., Tang Y., Dou K.-F., Tao K.-S. (2011). Spinal Toll like Receptor 3 Is Involved in Chronic Pancreatitis-Induced Mechanical Allodynia of Rat. Mol. Pain.

[69] Olesen S.S., Brock C., Krarup A.L., Funch–Jensen P., Arendt–Nielsen L., Wilder–Smith O.H., Drewes A.M. (2010). Descending Inhibitory Pain Modulation Is Impaired in Patients With Chronic Pancreatitis. Clin. Gastroenterol. Hepatol..

[70] Drewes A.M., Van Veldhuisen C.L., Bellin M.D., Besselink M.G., Bouwense S.A., Olesen S.S., Van Santvoort H., Vase L., Windsor J.A. (2021). Assessment of Pain Associated with Chronic Pancreatitis: An International Consensus Guideline. Pancreatology.

[71] Olesen S.S., Bouwense S.A.W., Wilder–Smith O.H.G., Van Goor H., Drewes A.M. (2011). Pregabalin Reduces Pain in Patients With Chronic Pancreatitis in a Randomized, Controlled Trial. Gastroenterology.

[72] Bouwense S.A.W., Olesen S.S., Drewes A.M., Poley J.-W., Van Goor H., Wilder-Smith O.H.G. (2012). Effects of Pregabalin on Central Sensitization in Patients with Chronic Pancreatitis in a Randomized, Controlled Trial. PLoS ONE.

[73] Drewes A.M., Frøkjær J.B., Olesen S.S., Singh V.K., Talukdar R., Windsor J.A. (2025). Pain in Chronic Pancreatitis: Navigating the Maze of Blocked Tubes and Tangled Wires. Gastroenterology.

[74] Liu Q., Ko C.-Y., Zheng C., Ye L., Liu B., Gao H., Huang D., Chou D. (2020). Decreased Glutamatergic Synaptic Strength in the Periaqueductal Gray Contributes to Maintenance of Visceral Pain in Male Rats with Experimental Pancreatitis. Neuroscience.

[75] Ren D., Li J.-N., Qiu X.-T., Wan F.-P., Wu Z.-Y., Fan B.-Y., Zhang M.-M., Chen T., Li H., Bai Y. (2022). Anterior Cingulate Cortex Mediates Hyperalgesia and Anxiety Induced by Chronic Pancreatitis in Rats. Neurosci. Bull..

[76] Hansen T.M., Muthulingam J.A., Drewes A.M., Olesen S.S., Frøkjær J.B. (2019). Cingulate Glutamate Levels Associate with Pain in Chronic Pancreatitis Patients. NeuroImage Clin..

[77] Wu J., Kuang W., Zhu Z., Dou J., Yao J., Cao J., Zhang F., Xu G. (2025). Upregulation of NR2B Subunits of NMDA Receptors in the Lateral Parabrachial Nucleus Contributes to Chronic Pancreatitis Pain. CNS Neurosci. Ther..

[78] Ceyhan G.O., Bergmann F., Kadihasanoglu M., Altintas B., Demir I.E., Hinz U., Müller M.W., Giese T., Büchler M.W., Giese N.A. (2009). Pancreatic Neuropathy and Neuropathic Pain—A Comprehensive Pathomorphological Study of 546 Cases. Gastroenterology.

[79] Zhu Y., Colak T., Shenoy M., Liu L., Pai R., Li C., Mehta K., Pasricha P.J. (2011). Nerve Growth Factor Modulates TRPV1 Expression and Function and Mediates Pain in Chronic Pancreatitis. Gastroenterology.

[80] Zhu Y., Mehta K., Li C., Xu G.-Y., Liu L., Colak T., Shenoy M., Pasricha P.J. (2012). Systemic Administration of Anti-NGF Increases A-Type Potassium Currents and Decreases Pancreatic Nociceptor Excitability in a Rat Model of Chronic Pancreatitis. Am. J. Physiol.-Gastrointest. Liver Physiol..

[81] Ceyhan G.O., Bergmann F., Kadihasanoglu M., Erkan M., Park W., Hinz U., Giese T., Muller M.W., Buchler M.W., Giese N.A. (2007). The Neurotrophic Factor Artemin Influences the Extent of Neural Damage and Growth in Chronic Pancreatitis. Gut.

[82] Demir I.E., Schorn S., Schremmer-Danninger E., Wang K., Kehl T., Giese N.A., Algül H., Friess H., Ceyhan G.O. (2013). Perineural Mast Cells Are Specifically Enriched in Pancreatic Neuritis and Neuropathic Pain in Pancreatic Cancer and Chronic Pancreatitis. PLoS ONE.

[83] Hoogerwerf W.A., Shenoy M., Winston J.H., Xiao S.-Y., He Z., Pasricha P.J. (2004). Trypsin Mediates Nociception via the Proteinase-Activated Receptor 2: A Potentially Novel Role in Pancreatic Pain. Gastroenterology.

[84] Hoogerwerf W.A., Gondesen K., Xiao S.-Y., Winston J.H., Willis W.D., Pasricha P.J. (2005). The Role of Mast Cells in the Pathogenesis of Pain in Chronic Pancreatitis. BMC Gastroenterol..

[85] Ceyhan G.O., Deucker S., Demir I.E., Erkan M., Schmelz M., Bergmann F., Müller M.W., Giese T., Büchler M.W., Giese N.A. (2009). Neural Fractalkine Expression Is Closely Linked to Pain and Pancreatic Neuritis in Human Chronic Pancreatitis. Lab. Investig..

[86] Demir I.E., Friess H., Ceyhan G.O. (2015). Neural Plasticity in Pancreatitis and Pancreatic Cancer. Nat. Rev. Gastroenterol. Hepatol..

[87] Zhu J., Miao X.-R., Tao K.-M., Zhu H., Liu Z.-Y., Yu D.-W., Chen Q.-B., Qiu H.-B., Lu Z.-J. (2017). Trypsin-Protease Activated Receptor-2 Signaling Contributes to Pancreatic Cancer Pain. Oncotarget.

[88] Friess H., Zhu Z.-W., Di Mola F.F., Kulli C., Graber H.U., Andren-Sandberg Å., Zimmermann A., Korc M., Reinshagen M., Büchler M.W. (1999). Nerve Growth Factor and Its High-Affinity Receptor in Chronic Pancreatitis. Ann. Surg..

[89] Pfizer Inc Safety and Efficacy of Tanezumab in Patients With Chronic Pancreatitis. ClinicalTrials.gov 2010, NCT01146561. NCT01146561.

[90] Roemer F.W., Hochberg M.C., Carrino J.A., Kompel A.J., Diaz L., Hayashi D., Crema M.D., Guermazi A. (2023). Role of Imaging for Eligibility and Safety of A-NGF Clinical Trials. Ther. Adv. Musculoskelet..

[91] Garami A., Shimansky Y.P., Rumbus Z., Vizin R.C.L., Farkas N., Hegyi J., Szakacs Z., Solymar M., Csenkey A., Chiche D.A. (2020). Hyperthermia Induced by Transient Receptor Potential Vanilloid-1 (TRPV1) Antagonists in Human Clinical Trials: Insights from Mathematical Modeling and Meta-Analysis. Pharmacol. Ther..

[92] AstraZeneca A Study of the Safety, Tolerability, and Pharmacokinetics of MEDI0618 in Healthy Volunteers. ClinicalTrials.gov 2019, NCT04198558. NCT04198558.

[93] Novakand Pharma AB Safety, Tolerability and Pharmacokinetics After Continuous Infusion of KAND567. ClinicalTrials.gov 2023, NCT06030375. NCT06030375.

[94] Drewes A.M., Bouwense S.A.W., Campbell C.M., Ceyhan G.O., Delhaye M., Demir I.E., Garg P.K., Van Goor H., Halloran C., Isaji S. (2017). Guidelines for the Understanding and Management of Pain in Chronic Pancreatitis. Pancreatology.

